# Innovative applications and research advances of bacterial biosensors in medicine

**DOI:** 10.3389/fmicb.2025.1507491

**Published:** 2025-04-23

**Authors:** Mengting Liu, Wenjie Yang, Wenqi Zhu, Daojun Yu

**Affiliations:** ^1^The Fourth School of Clinical Medicine, Zhejiang Chinese Medical University (Hangzhou First People’s Hospital), Hangzhou, China; ^2^Affiliated Hangzhou First People’s Hospital, Westlake University School of Medicine, Hangzhou, China

**Keywords:** bacteria, biosensors, disease diagnosis, personalized medicine, real-time monitoring

## Abstract

The demand for early disease detection, treatment monitoring, and personalized medicine is increasing, making it more imperative than ever to create effective, accurate, portable, intelligent, multifunctional diagnostic equipment. Bacteria possess a remarkable perception of their surroundings and have the capacity to adapt by altering the expression of specific genes. Bacteria interact with target substances and produce detectable signals in response to their presence or concentration. This unique property has been harnessed in the development of bacterial biosensors. Due to groundbreaking advancements in synthetic biology, genetic engineering now enables the creation of bacteria tailored with exceptional detecting traits. In addition to meeting a wide range of application needs, this allows quick and precise detection in intricate settings and offers a strong technological basis for early disease diagnosis and treatment monitoring. This article reviews the applications and recent advancements of bacterial biosensors in the medical field and discusses the challenges and obstacles that remain in their research and application.

## 1 Introduction

With the rapid advancement of biotechnology and growing demands for affordable healthcare, particularly in resource limited settings, traditional diagnostic methods reliant on complex instrumentation such as PCR and mass spectrometry face challenges in achieving rapid, sensitive, and portable testing solutions (Law et al., 2015). Bacterial biosensors, which employ engineered bacteria as programmable sensing elements, offer a cost effective and scalable alternative. These devices detect target analytes including pathogens, metabolites and biomolecules, through synthetic genetic circuits, converting biological signals into quantifiable outputs such as electrical currents or fluorescence via electrochemical or optical interfaces (Nakamura et al., 2008). Recent advances in synthetic biology, such as CRISPR-based gene circuits and synthetic circuit, have enabled precise tuning of bacterial sensing pathways. Such innovations expand medical applications from early disease diagnosis to personalized therapeutics (Riglar and Silver, 2018). However, critical barriers such as biosafety concerns and functional stability hinder clinical translation. This article broadly examines recent advances in bacterial biosensor design, highlights their emerging roles in precision medicine, and critically discusses unresolved technical and regulatory hurdles.

## 2 Principles and mechanisms of bacterial biosensors

Bacteria possess remarkable environmental adaptability, allowing them to detect and react to environmental alterations, such as variations in chemical concentrations, temperature, and pH levels (Andrianantoandro et al., 2006; Park et al., 2013). This unique trait makes bacteria one of the most suitable biological recognition components for biosensors (Soltani Zarrin et al., 2018). Bacterial biosensors achieve specific detection of target molecules or environmental factors by converting biological responses into quantifiable signals. Their functionality relies on the coordinated operation of three core components: the input module (sensing unit), signal transduction module (processing unit), and output module (response unit) (Chen et al., 2023). The input module of bacterial biosensors functions as the sensing element responsible for the specific recognition of and response to target signals, relying on selective molecular interactions between biomolecules (Saltepe and Kehribar et al., 2018). This module employs both naturally occurring components such as transcription factors and membrane receptors and engineered constructs, including aptamers or nucleic acid switches, to directly capture external stimuli (e.g., chemical signals, physical cues, or biomarkers) and convert them into intracellular signals amenable to processing (Ding et al., 2021). Upon target recognition, the sensing elements activate the signal transduction module through distinct triggering mechanisms: conformational changes, induced dimerization, conditional stabilization, or enzymatic reactions (Miller et al., 2022). For example, conformational changes occur when transcription factors or membrane receptors bind target molecules, inducing structural rearrangements that initiate downstream responses (Serganov and Nudler, 2013; Zhong et al., 2016); induced dimerization involves the binding of two monomeric molecules under specific conditions to form a functional dimer, thereby activating signaling pathways (Capra and Laub, 2012; Lazar and Tabor, 2021); conditional stabilization refers to molecular stability regulated by environmental parameters such as temperature (Schuster and Greenberg, 2008); and enzymatic amplification leverages enzyme-catalyzed substrate to product conversions to enhance detection sensitivity (Miller et al., 2022). Acting as the central hub, the signal transduction module bridges the input and output modules by transforming initial detection signals into processable intracellular signals while enabling amplification, integration, or logical operations. Natural bacterial systems predominantly utilize pathways such as two-component systems (TCS), in which histidine kinases (HK) recognize extracellular signals via their sensor domains, undergo autophosphorylation at histidine residues, and transfer phosphate groups to aspartate residues on response regulators (RR), thereby activating RR’s DNA-binding or enzymatic functions to regulate gene expression (Lazar and Tabor, 2021; Salis et al., 2009; Tanna et al., 2021); quorum sensing (QS), a density-dependent communication mechanism mediated by autoinducers (AIs) that accumulate to threshold concentrations to trigger QS-regulated behaviors such as bioluminescence, virulence factor production, or biofilm formation (Kumari et al., 2006; Lee et al., 2013); and chemotaxis systems, which direct bacterial motility toward nutrient-rich environments or away from harmful substances (Karmakar, 2021). The output module translates processed intracellular signals into detectable and quantifiable physical, chemical, or biological responses (Lopreside et al., 2019). Optical outputs such as fluorescence (green fluorescent protein, GFP) or bioluminescence (luciferase) rely on promoter-driven reporter gene expression, where fluorescence intensity correlates linearly with target concentration to achieve high-sensitivity detection (Belkin, 2003; Roda et al., 2011; van der Meer and Belkin, 2010). Chromogenic outputs exploit enzymatic cleavage of substrates, including X-gal hydrolysis by lacZ-encoded β-galactosidase to produce a blue chromogen (Mascher et al., 2004), enabling semi-quantitative visual or spectrophotometric analysis without specialized equipment, a feature particularly advantageous for point-of-care or resource-limited settings. Electrochemical outputs detect target-induced changes in redox reactions, ion concentrations, or charge distributions at electrode surfaces, with signals quantified through current, voltage, or impedance measurements (Johnson et al., 2017; Zhu et al., 2023). Collectively, these modular frameworks enable bacterial biosensors to address diverse biomedical challenges by balancing sensitivity, specificity, and practicality for applications spanning diagnostics, environmental monitoring, and therapeutic evaluation.

**FIGURE 1:**
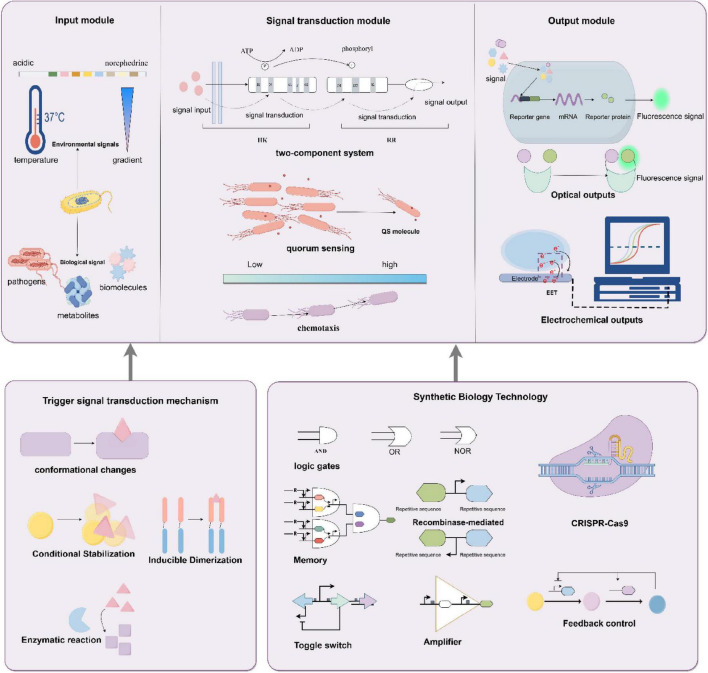
Principles and mechanisms of bacterial biosensors. The input module receives environmental signals (e.g., temperature gradients, acidic environments) and biological signals (e.g., pathogens, metabolites), triggering signal transduction through mechanisms such as conformational changes, conditional stabilization, or enzymatic reactions. The signal transduction module, which comprises two-component systems, quorum sensing, and chemotaxis, processes these signals, while the output module converts them into optical (e.g., fluorescence) or electrochemical responses. Synthetic biology techniques (e.g., logic gates, memory switches, feedback regulation, and CRISPR-Cas9 gene editing) further optimize the sensor’s detection sensitivity and dynamic response range. By Figdraw.

Synthetic biology holds significant potential to advance the development of bacterial biosensors. A cornerstone of this advancement lies in the strategic deployment of gene-editing tools, particularly CRISPR-Cas9 technology (Doudna and Charpentier, 2017). By enabling targeted knockout of genes responsible for non-specific responses or background interference, CRISPR-Cas9 enhances sensor specificity through noise reduction (de la Fuente-Núñez and Lu, 2017). Complementarily, gene knock-in techniques integrate functional genetic elements to amplify both sensitivity and specificity, permitting reliable detection even at ultralow target concentrations. The redesign of endogenous signaling circuits constitutes another critical strategy (Jung et al., 2018). Native bacterial signal transduction pathways, composed of receptor proteins, transcription factors, and effector proteins, are systematically engineered to maintain their intrinsic efficiency while enhancing analytical versatility (Jung et al., 2018; Raut et al., 2013; Skjoedt et al., 2016). For example, modifying receptor binding sites to accommodate structurally analogous targets expands the detectable analyte spectrum, thereby addressing diverse diagnostic needs. Synthetic genetic circuit construction further introduces novel functionalities (Sedlmayer et al., 2018; Wang et al., 2013). Modular components, including AND, OR, and NOR logic gates, enable coordinated multi-signal processing, allowing biosensors to function with high precision in complex matrices (Bonnet et al., 2013; Tang et al., 2021; Wang et al., 2011). Memory modules such as transcription factor-based toggle switches and recombinase-mediated memory circuits confer bacteria with programmable “memory storage,” recording prior exposure to specific analytes (Riglar et al., 2017). This capability facilitates accelerated, context-dependent responses upon re-exposure, which is essential for monitoring dynamic fluctuations in environmental conditions or disease biomarkers (Archer et al., 2012; Park et al., 2013). To optimize performance, circuit design integrates signal amplification coupled with feedback control mechanisms. Positive feedback loops serve as biological amplifiers (Wan et al., 2019), heightening sensitivity to low-abundance signals, whereas negative feedback mechanisms act as stabilizers, preventing signal oversaturation and preserving cellular homeostasis (Jia et al., 2019). This dual regulatory framework ensures sustained operational stability across prolonged and variable detection scenarios (Tiwari et al., 2013). Collectively, these innovations position synthetic biology-driven bacterial biosensors as robust tools for applications spanning environmental monitoring to clinical diagnostics Figure 1.

## 3 Applications of bacterial biosensors in disease detection and treatment monitoring

Bacterial biosensors are an innovative biotechnological tool with significant potential across various fields (Plotnikova et al., 2016). In environmental monitoring, they are used to detect organic pollutants, heavy metal ions, and other harmful substances in soil and water (Plotnikova et al., 2016). These sensors employ engineered bacteria to generate specific biological reactions to pollutants, which are then converted into measurable signals (Coelho et al., 2015). For instance, bacterial biosensors can detect organic pollutants like phenolic compounds and polycyclic aromatic hydrocarbons (Plotnikova et al., 2016), as well as heavy metals such as mercury (Chen et al., 2020), arsenic (Hui et al., 2023), and chromium (Francisco et al., 2019). Their high sensitivity and selectivity enable rapid detection and early warning of environmental pollutants. In food safety, bacterial biosensors offer a faster, more cost-effective alternative to traditional methods (Gao et al., 2023), detecting harmful substances like antibiotics, hydrogen peroxide, pesticide residues, and pathogenic bacteria (Poikulainen et al., 2020). These sensors allow for quick screening and early warning, ensuring food safety and reliability (Liao et al., 2006). Additionally, bacterial biosensors are gaining attention in the medical field for their role in diagnosing gastrointestinal diseases, cancer, and other applications Table 1. We will now explore their specific uses and potential value in these areas.

**TABLE 1:** Application of bacterial biosensors in medicine.

Input target	Engineered bacterial strain	Mechanism	Report gene	Associated disease	References
N-acyl homoserine lactones (AHLs)	*E.coli* JM109	Quorum sensing (QS)	luxCDABE (luciferase)	Gastrointestinal diseases	Kumari et al., 2006
Autoinducer-2 (AI-2)	*Vibrio harveyi BB170*	Quorum sensing	luxCDABE (luciferase)	Gastrointestinal diseases	Raut et al., 2013
Tetrathionate	*E. coli* NGF-1	Synthetic biology	cI/Cro memory switch and lacZ reporter	Gastrointestinal diseases	Kotula et al., 2014
Thiosulfate	*E. coli Nissle 1917*	Two-component system	Fluorescence	Gastrointestinal diseases	Riglar et al., 2017
Thiosulfate	*E. coli Nissle 1917*	Two-component system	Fluorescence	Gastrointestinal diseases	Daeffler et al., 2017
Nitric Oxide (NO)	*E. coli Nissle 1917*	Synthetic circuit	Fluorescence (GFP)	Gut inflammation	McKay et al., 2018
Nitric Oxide	*E. coli Nissle 1917*	Positive feedback circuit	Fluorescence (GFP)	Gut inflammation	Chen et al., 2021
Lactate	*E. coli Nissle 1917*	Synthetic biology	lacZ (β-galactosidase) and luxCDABE (luciferase)	Liver metastases	Danino et al., 2015
Tumor microenvironment	*Salmonella Typhimurium* ΔppGpp	ChemotaxisSynthetic circuit	ClyA (oncolytic protein) and LuxCDABE (luciferase)	Colon/liver cancer models	Nguyen et al., 2010
Tumor microenvironment	*Salmonella Typhimurium*	Chemotaxis Synthetic circuit	RLuc8 (Renilla luciferase) and ClyA (therapeutic gene)	Colon/liver cancer models	Jiang et al., 2013
Tumor microenvironment	*Salmonella VNP20009* (ΔmsbB)	Chemotaxis Synthetic circuit	Dual-channel imaging	Pancreatic ductal adenocarcinoma (PDAC)	Zhou et al., 2016
Mutant KRAS DNA	*Acinetobacter baylyi*	Chemotaxis	Fluorescence (GFP) and Kanamycin resistance gene	Gastric/colorectal cancer detection	Cooper et al., 2023
Hypoxia/ATP	*Salmonella Typhimurium* Δ*ppGpp*	Chemotaxis	RLuc8 (Renilla luciferase)	Myocardial Infarction	Le et al., 2011
Urinary glucose	*E. coli*	Synthetic circuit	Fluorescence (GFP/RFP)	Diabetes mellitus	Courbet et al., 2015
Cytarabine (Ara-C)	*E. coli MG1655*Δ*cdd*	Synthetic circuit	luxCDABE (luciferase)	Leukemia	Alloush et al., 2010
CAI-1	*E. coli*	Quorum sensing and synthetic circuit	Fluorescence(GFP)	Cholera	Holowko et al., 2016
Heme	*E. coli Nissle 1917*	Synthetic circuit	luxCDABE (luciferase)	Gastrointestinal diseases and Iron-related disorders	Bhatt, 2018
Multiple inflammation mediators	*E. coli Nissle 1917*	Synthetic circuit	luxCDABE (luciferase) wireless transmission	Gastrointestinal diseases	Inda-Webb et al., 2023
Bile acids	*E. coli Nissle 1917*	Modularized receptor	lacZ (β-galactosidase)	Liver disease, post-transplant monitoring	Chang et al., 2018; Chang et al., 2021
Skin inflammation	*Staphylococcus epidermidis*	Innate immunomodulation and bioelectronic sensing	Wireless skin impedance/temperature data	Psoriasis, inflammatory skin diseases	Shi et al., 2024
Lactate	*Shewanella oneidensis MR-1*	Extracellular electron transport (EET)	Electrochemical signal	Cancer	Wang et al., 2022

### 3.1 Applications of bacterial biosensors in gastrointestinal diseases

**FIGURE 2:**
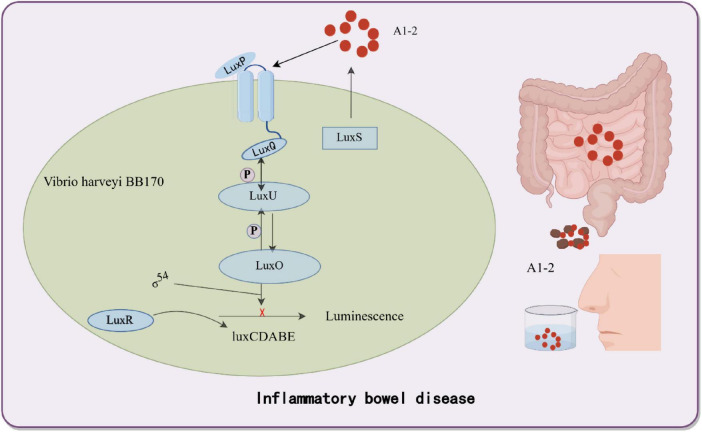
Bacterial biosensor based on a quorum sensing system for inflammatory bowel disease detection. Utilizing the quorum sensing system of *Vibrio harveyi BB170*, this biosensor detects the inflammatory bowel disease (IBD)-associated autoinducer AI-2, triggering the expression of bioluminescent genes to generate quantitatively detectable fluorescent signals. By Figdraw.

Inflammatory bowel disease (IBD) is a chronic inflammatory disorder driven by genetic susceptibility, immune dysregulation, and gut microbiota dysbiosis (Bourgonje et al., 2020; Reese et al., 2018; York, 2023). Quorum sensing (QS), a bacterial communication mechanism mediated by signaling molecules such as AI-2, AHLs, and AIP, plays a dual role in gut homeostasis (Ge et al., 2020; Raut et al., 2013). Early studies established the clinical relevance of QS molecules in gastrointestinal diseases. For instance, Kumari et al. engineered *E. coli* JM109 as a biosensor by introducing *Pseudomonas aeruginosa* QS regulatory systems (LasR/RhlR) through plasmids pSB406 and pSB1075, coupled with the *luxCDABE* bioluminescent reporter. This system enabled sensitive detection of *N*-acyl homoserine lactones (AHLs) in human saliva and stool samples, achieving a detection limit of 1 × 10^–9^ M without extensive sample preparation. Their work demonstrated that AHLs are present in both healthy individuals and Crohn’s disease patients, with levels correlating to microbial dysbiosis (Kumari et al., 2006, 2008). The use of *E. coli* as a chassis organism highlighted its adaptability in heterologous QS circuit engineering, though the role of AHLs in IBD-associated inflammation remained to be fully elucidated. While QS coordinates beneficial microbial interactions, its dysregulation may promote pathogenic behaviors and amplify inflammation in IBD (Chang, 2015; Zhang J. et al., 2023). To leverage QS for IBD monitoring, Nilesh Raut’s team developed a biosensor using *Vibrio harveyi BB170*. In this system, AI-2 binds to the cytoplasmic receptor LuxP, triggering a phosphorylation cascade (LuxQ→ LuxU→ LuxO) that activates the *luxCDABE* promoter, producing bioluminescence proportional to AI-2 concentration (Kumari et al., 2006; Raut et al., 2013; Figure 2). Feces provide limited spatiotemporal resolution for monitoring intestinal inflammation, despite reflecting gut microbiota dynamics (Chang, 2015; Naydich et al., 2019). To overcome this, synthetic biology strategies deploy engineered bacteria as living biosensors capable of *in situ* signal recording (Chang, 2015; Naydich et al., 2019). Kotula et al. (2014) designed and constructed a two-part system that comprised a “trigger element” and a “memory element.” Kotula et al.’s (2014) team pioneered a two-component memory system in *E. coli*, comprising a trigger element (tetracycline-responsive promoter) and a λ phage-derived cI/Cro memory switch. In tetracycline-treated mice, the trigger activated Cro expression, irreversibly switching the bacterial state from cI (silent) to Cro (active), which persisted for ≥ 5 days without inducer (Burrill et al., 2012; Kotula et al., 2014; Moon et al., 2012). This system laid the foundation for chronic gut monitoring. Thiosulfate and tetrathionate is a transient product of reactive oxygen species (ROS), which are produced during inflammation (Winter and Bäumler, 2014; Winter et al., 2013). Expanding on this, Riglar et al. (2017) integrated the *Salmonella Typhimurium P_*ttrBCA*_* promoter (responsive to tetrathionate) with the λ memory module and lacZ reporter in *E. coli NGF-1*. Upon detecting tetrathionate, β-galactosidase hydrolyzed X-gal to generate blue fecal colonies. Remarkably, this sensor functioned for > 6 months in mice, demonstrating sustained *in vivo* operation (Riglar et al., 2017). In parallel, Daeffler et al. (2017) engineered an *E. coli Nissle 1917* biosensor using a *Shewanella* oneidensis-derived two-component system (TsrA/TsrR) to detect thiosulfate, a biomarker of gut inflammation. The sensor activates a fluorescent reporter via phosphorylation cascades, achieving a detection limit of 50 μM within 2 h. In murine colitis models, thiosulfate levels correlated strongly with histopathological scores (r = 0.68, *p* < 0.01) (Daeffler et al., 2017). These advances demonstrate the potential of synthetic biology in developing multiplexed, long-term gut surveillance systems capable of non-invasively tracking dynamic inflammatory processes.

The interactions between the gut microbiota and the human host, as well as changes in its metabolic products, have been linked to a variety of pathological conditions, such as metabolic disorders, immunological diseases, cancer, neurological diseases, and behavioral disorders (Pesce et al., 2022). Bacterial biosensors represent non-invasive instruments pivotal in intestinal health monitoring, capable of detecting intestinal metabolites and translating these signals into readily observable outcomes, encompassing colorimetric changes, fluorescence emission, and electrical signals, thereby facilitating accurate assessment of gut health status (Pesce et al., 2022; Winter et al., 2010). Transient molecules in the gastrointestinal system, such as nitric oxide (NO) and hydrogen sulfide (H2S), are critical but elusive inflammation markers due to their short half-life and high reactivity (Riglar et al., 2017). At the start, To address NO detection challenges, McKay et al. (2018) engineered a dual-plasmid system in *E. coli Nissle 1917*. The first plasmid contains a NO-sensitive promoter (PnorV) driving T7 RNA polymerase (T7Pol) expression, while the second plasmid utilizes a T7/lac hybrid promoter to control green fluorescent protein (GFP) expression. This cascade amplification enables visualization of gut NO levels with a detection limit of 10 nM within 1 h, validated in murine models (McKay et al., 2018). Subsequently, Chen et al.’s (2021) team enhanced sensitivity by designing a positive feedback circuit, with the NO-responsive transcription factor NorR activates its own expression alongside superfolder GFP (sfGFP) under the PnorV promoter. Elevated NO concentrations trigger NorR self-amplification, reducing the detection threshold to 2 nM and accelerating response time by 3-fold (Chen et al., 2021). These ingestible biosensors exemplify the potential of synthetic biology in real-time gut monitoring.

### 3.2 Applications of bacterial biosensors in cancer

**FIGURE 3:**
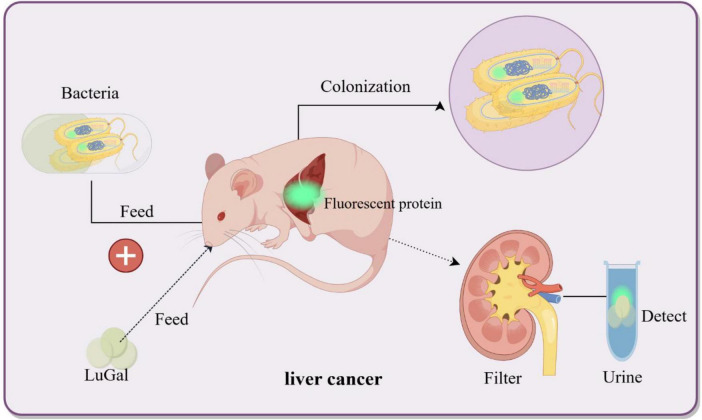
Bacterial biosensors for non-invasive detection of liver cancer. Genetically engineered bacteria are designed to colonize the liver cancer microenvironment and induce the expression of fluorescent proteins upon sensing specific biomarkers (e.g., LuGal). The fluorescent molecules are excreted via the bloodstream into urine, thereby enabling non-invasive detection of liver cancer biomarkers. By Figdraw.

Cancer is a leading cause of global mortality, with liver metastases occurring in > 50% of patients with gastrointestinal malignancies and associated with a 5 years survival rate < 15% due to delayed detection (Groen, 1999; Liang et al., 2022). Current interventions (surgical resection, radiotherapy, etc.) target macroscopic lesions (> 1 cm) but fail to eliminate micrometastases (< 1 mm), which evade detection by conventional imaging (CT/MRI) and seed recurrent tumors (Chien et al., 2021; Schroeder et al., 2011). To address this, bacterial biosensors engineered as “living diagnostics” have emerged. In engineered *E. coli Nissle 1917*, synthetic genetic circuits employ lactate-responsive promoters to drive tumor-specific expression of β-galactosidase (lacZ) and bioluminescent reporters (luxCDABE) (Meighen, 1991). The oral delivery strategy leverages the gut-liver axis, through which EcN crosses the intestinal barrier via bile acid transporters and colonizes hepatic metastases within 24 h, thereby avoiding systemic toxicity (Chien et al., 2021). In preclinical models, the engineered EcN produced bioluminescence, enabling longitudinal imaging of liver metastases and improving visualization of metastatic lesions. Additionally, in mice fed with these probiotics, the bacteria colonized liver tumors specifically within 24 h while being cleared from healthy organs. Upon confirming colonization, LuGal (D-luciferin-O-β-galactoside) was administered. The β-galactosidase produced by EcN hydrolyzes LuGal into luciferin, which is subsequently excreted in urine. A complementary urinary detection system achieved rapid diagnosis using only 1 μL of urine, with detectable signals within 24 h post-administration. Remarkably, no adverse effects on mouse health were observed during 12 months of monitoring (Danino et al., 2015; Schultz et al., 2005; Figure 3). Despite promising sensitivity, clinical translation requires optimization of bacterial containment strategies to prevent horizontal gene transfer and validation in human trials. Genetically engineered Salmonella enterica serovar *Typhimurium* ΔppGpp exhibits tumor-specific colonization by exploiting the nutrient-rich tumor microenvironment (TME), making it a promising platform for theranostic applications (Nguyen and Min, 2017). Nguyen et al. (2010) engineered this strain to co-express cytolysin A (ClyA) and bacterial luciferase *(LuxCDABE*) under the control of an L-arabinose-inducible PBAD promoter. In murine colon and liver cancer models, intravenous administration led to selective tumor colonization, with oral L-arabinose triggering ClyA-mediated tumor lysis (62% volume reduction, *p* < 0.01) and intraperitoneal D-luciferin enabling real-time tracking imaging (R^2^ = 0.85 vs. tumor burden) (Nguyen et al., 2010). To enhance spatiotemporal control, Jiang’s et al. (2013) team integrated a Tet-On system, where tetracycline dose-dependently activates *RLuc8* (reporter) and ClyA (therapeutic gene) through Ptet promoters. This dual-function design allowed simultaneous imaging-guided therapy: RLuc8 bioluminescence correlated with ClyA efficacy (30% apoptosis increase per 1 μg/mL tetracycline), enabling personalized dosing (Jiang et al., 2013).

Pancreatic ductal adenocarcinoma (PDAC), characterized by its aggressive nature and poor prognosis (5 years survival < 10%), remains a major clinical challenge despite advancements in multimodal therapies (Principe et al., 2017). To address this, Zhou et al. (2016) engineered an attenuated *Salmonella strain (VNP20009)* through deletion of endotoxin-producing genes (msbB), enhancing its safety profile while preserving tumor-targeting capability (Low et al., 2004). This modified strain was designed as a theranostic agent, combining therapeutic potential with real-time diagnostic imaging. In their study, human PDAC cells (CFPAC-1) stably expressing the far-red fluorescent protein mKate2 (λem ≈ 635 nm) were implanted in mice to establish tumors (Piatkevich et al., 2010). Following intratumoral injection, the engineered Salmonella expressing bacterial luciferase (*luxCDABE)* colonized the tumor microenvironment, enabling dual-channel tracking via the IVIS Spectral Imaging System (Cheng et al., 2014; Toso et al., 2002). The distinct emission spectra of mKate2 (minimizing tissue autofluorescence) and luciferase (λem ≈ 490 nm) allowed simultaneous quantification of tumor burden and bacterial localization. Through longitudinal imaging, researchers mapped tumor growth kinetics and evaluated the spatiotemporal distribution of co-administered chemotherapeutics, demonstrating a correlation between bacterial density and drug efficacy (Hiddemann and Büchner, 2001; Zhou et al., 2016). This integrated approach highlights the potential of bacterial-based theranostics to refine PDAC management, though clinical translation requires further validation of safety and targeting specificity. Horizontal gene transfer (HGT) is the process by which organisms transfer genetic material to other cells rather than their offspring, in contrast to vertical transmission. HGT happens often in microorganisms and between prokaryotes and eukaryotes (Mell and Redfield, 2014; Soucy et al., 2015). However, the broad field of bacteria utilizing HGT mechanisms to detect mammals and respond to their DNA remains largely unexplored. Cooper’s et al. (2023) research team proposed the Cellular Assay for Targeted CRISPR-discriminated Horizontal Gene Transfer (CATCH), which is based on this principle. This technique integrates target DNA into the genome of engineered *Acinetobacter baylyi*, which functions as a biosensor to identify certain extracellular DNA. The KRAS gene is a typical oncogene. It controls cell growth normally, but when it is mutated, it causes uncontrolled cell proliferation and dysregulation (Priestley et al., 2019; Vogelstein et al., 1988). To eliminate false positive results produced by natural KRAS binding, the CRISPR technique efficiently addresses this issue. The CRISPR-Cas system was designed as a bacterial defensive mechanism for cutting foreign DNA, but it can be purposefully altered to modify its cutting positions and targets. The CATCH technique uses a specially designed sgRNA as a guide to accurately target the DNA sequence through complementary pairing. The CRISPR-Cas system is specifically designed to cut just the mutant KRAS gene that lacks a specific PAM sequence, with no effect on the normal KRAS gene (Cooper and Hasty, 2020). By integrating the CRISPR-Cas system and reporter genes (GFP or kanamycin resistance genes) into bacteria, the novel bacterial biosensor may co-culture with tumor cells in a variety of complex situations. The detection of the target DNA is demonstrated by the monitoring of GFP expression and bacterial growth in the kanamycin-selective media (Cooper et al., 2023). In the future, such engineered bacteria will have limitless potential for detecting cancer and precancerous lesions, and they are expected to help in the prevention of stomach and colorectal cancer. It is also possible to further modify these bacterial biosensors to create novel detectors with a variety of detecting goals in mind.

### 3.3 Applications of bacterial biosensors in myocardial infarction

Myocardial infarction (MI), characterized by coronary artery occlusion leading to ischemic necrosis of cardiac tissue, remains a leading cause of global mortality (Tsao et al., 2023). The auxotrophic *Salmonella Typhimurium* Δ*ppGpp* strain exhibits selective tropism to infarcted myocardium, driven by hypoxia-responsive chemotaxis and necrotic cell-derived ATP gradients (Yi et al., 2020). To enable real-time imaging, this strain was engineered with a RLuc8 construct, where the *E. coli* L-arabinose-inducible promoter strictly controls expression of Renilla luciferase variant RLuc8. Upon systemic L-arabinose administration, RLuc8 is activated exclusively in bacteria colonizing ischemic regions, generating localized bioluminescence signals detectable within 2 h post-injection (Loening et al., 2006). In murine MI models, this approach achieved a spatial resolution of 0.5 mm, identifying subendocardial infarcts comprising as little as 3% of left ventricular mass—a significant improvement over SPECT’s 10% threshold (Le et al., 2011). Importantly, intravenous delivery of Δ*ppGpp Salmonella* induced minimal systemic inflammation and no histopathological evidence of myocardial damage, underscoring its biosafety (Chang et al., 2021).

### 3.4 Applications of bacterial biosensors in diabetes mellitus

Diabetes mellitus, characterized by chronic hyperglycemia, requires rigorous glucose monitoring to prevent complications (Aloraynan et al., 2022). While fingerstick tests and continuous glucose monitors (CGMs) remain clinical standards, their invasiveness and cost drive demand for alternative methods (Liu et al., 2020). Urinary glucose (glycosuria) serves as a non-invasive proxy for hyperglycemia, though its utility is limited by a 1–2 h lag behind blood glucose levels and inter-individual renal threshold variations (de Sousa Vieira et al., 2022). To address this, Courbet et al. (2015) engineered *E. coli* to detect urinary glucose via a synthetic cpxP promoter—a stress-responsive element repurposed to activate GFP/RFP expression upon glucose uptake. The bacteria were encapsulated in alginate-PVA hydrogel beads, maintaining 90% fluorescence stability over 72 h in urine while preventing bacterial leakage (Harpaz et al., 2023). A genetic AND gate circuit further enhanced specificity: simultaneous glucose detection and hypoxia (mimicking bladder conditions) triggered GFP expression, achieving a detection limit of 0.1 mM glucose (equivalent to blood glucose ∼180 mg/dL) with 88.9% sensitivity and 96.3% specificity in diabetic urine samples (*n* = 150) (Courbet et al., 2015). Despite these advances, urinary glucose monitoring cannot replace real-time blood measurements due to physiological lag.

### 3.5 Applications of bacterial biosensors in monitoring cytarabine in leukemia

Bacterial biosensors have emerged as transformative tools for rapid drug sensitivity testing, particularly in predicting leukemia patients’ responses to cytarabine (Ara-C) (Hiddemann and Büchner, 2001). A key innovation is the engineering of an *E. coli MG1655* cytidine deaminase-deficient mutant (Δcdd), which cannot metabolize Ara-C to its inactive form (Ara-U). This strain was integrated with the *luxCDABE* operon to generate a bioluminescent reporter system responsive to intracellular Ara-CTP levels—the active metabolite of Ara-C that inhibits DNA polymerase α and induces leukemic cell death (Wang et al., 1998; Yamauchi et al., 2009). In co-culture assays with patient-derived leukemic cells, the biosensor quantifies bioluminescence intensity, which correlates with Ara-CTP accumulation and drug efficacy. Compared to traditional MTT assays requiring 3–5 days, this system delivers results within 8 h, achieving 85% sensitivity and 92% specificity in identifying Ara-C-resistant patients (*n* = 50) (Yamauchi et al., 2009). The Δcdd mutation ensures bacterial viability by preventing Ara-C detoxification, enabling continuous signal generation without interference from host cell metabolites (Alloush et al., 2010). By enabling rapid, low-cost drug sensitivity testing, this biosensor platform exemplifies the potential of synthetic biology to bridge precision medicine and global health accessibility, particularly in resource-limited settings.

### 3.6 Applications of bacterial biosensors in cholera

Cholera, caused by toxigenic *Vibrio cholerae*, demands rapid diagnostics to curb its high transmission risk. Conventional methods like culture enrichment require > 24 h and lack sensitivity (Rafique et al., 2016). Synthetic biology offers innovative solutions: Holowko et al. (2016) engineered non-pathogenic *E. coli* to detect *V. cholerae*-specific CAI-1 (10 nM detection limit) by integrating its QS system (CqsS sensor kinase and response regulators) with a CRISPRi-based genetic inverter. In this system, dCas9 represses GFP expression in the absence of CAI-1, while CAI-1 binding relieves repression, enabling fluorescence readout within 2 h—100-fold faster than ELISA (Duan and March, 2010; Holowko et al., 2016; Ng et al., 2011; Yamasaki et al., 2017). Parallelly, Mao et al. (2018) developed a probiotic *Lactococcus lactis* biosensor using a TetR-regulated mCherry reporter. CAI-1 inactivates TetR via allosteric displacement, inducing a 60-fold mCherry increase. For field applications, they replaced fluorescence with β-lactamase secretion: hydrolysis of nitrocefin triggers a yellow-to-red color shift in 15 min, achieving 95% concordance with PCR in clinical stool samples (Mao et al., 2018). Beyond detection, Jayaraman et al. (2017) engineered a “sense-and-kill” *E. coli* that secretes Art-085 lysin via the YebF pathway upon CAI-1 detection. In murine models, this system reduced intestinal *V. cholerae* loads by 3 logs within 6 h, outperforming oral rehydration alone (Jayaraman et al., 2017).

## 4 Applications of bacterial biosensors in personalized medicine

Personalized medicine aims to revolutionize healthcare by tailoring diagnostic and therapeutic strategies to individual patients through real-time, dynamic monitoring of biomarkers. For instance, IBD patients require frequent monitoring of intestinal inflammation to optimize anti-inflammatory therapies, yet current techniques cannot provide continuous, non-invasive insights into biomarker dynamics. Bacterial biosensors, engineered to detect specific molecules *in situ*, offer a transformative solution (Neurath, 2017). Bhatt (2018) developed ingestible microbial electronic devices (IMBED) for personalized gut monitoring. They engineered *E. coli Nissle 1917* to express heme-responsive genetic circuits (Phas promoter) and the *luxCDABE* operon, enabling bioluminescence upon heme detection. The IMBED encapsulates bacteria in a chamber with a nanoporous membrane (pore size < 50 nm), allowing metabolite influx while preventing bacterial escape (Liu et al., 2021). A silicon photodetector converts bioluminescence into wireless signals transmitted to smartphones, enabling real-time tracking of gut biomarkers (Mimee et al., 2018). In murine models, IMBED achieved a heme detection limit of 1 μM within 30 min, demonstrating potential for personalized management of iron-related disorders (Bhatt, 2018). However, IMBED requires enteric coating to neutralize gastric acid, and its long-term reliability may be compromised by intestinal peristalsis or biofilm formation. Future iterations could integrate pH-resistant circuits and anti-fouling membranes to enhance clinical viability. Recent advances in bacterial biosensors have enabled real-time tracking of transient gastrointestinal molecules for personalized medicine. Inda-Webb et al. (2023) engineered an ingestible electronic capsule (< 1.4 cm^3^) integrating *E. coli Nissle 1917* biosensors with silicon photodiode arrays. The bacteria were modified to express engineered sensing proteins and recombinase-based memory circuits, allowing continuous recording of oxidative stress markers. A low-power module wirelessly transmits bioluminescent signals (triggered by luxCDABE promoter) to smartphones, achieving real-time monitoring of multiple inflammation mediators, including thiosulfate, tetrathionate, hydrogen peroxide (H2O2), and nitric oxide (NO). For instance, the system detects H2O2 with a limit of 10 nM and responds within < 5 min, enabling dynamic tracking of redox imbalance during disease progression. The capsule, validated in porcine colitis models, demonstrated a direct correlation between H2O2 concentration spikes and disease flare severity, enabling data-driven adjustments to antioxidant therapies (Inda-Webb et al., 2023; Liu et al., 2023). This platform represents a closed-loop theranostic system that integrates biomarker detection, therapeutic decision-making, and treatment response monitoring, thereby advancing non-invasive and individualized approaches to gastrointestinal care. MeRALD (Engineered Modularized Receptors Activated by Ligand-induced Dimerization) platform to address critical gaps in liver disease management (Chang et al., 2018). By engineering *E. coli Nissle 1917* with programmable receptors sensitive to bile acids, their system detects pathological concentrations of these hepatic biomarkers through ligand-induced dimerization mechanisms (Chang et al., 2021). When bile acids bind to the modularized TcpP18 receptor, conformational changes trigger β-galactosidase expression via a LacZ reporter, generating quantifiable colorimetric signals in fecal samples (Abdelbasset et al., 2023). This innovation demonstrated clinical utility in liver transplant monitoring, while enabling smartphone-based color analysis for home testing. Notably, the platform’s modular design allows rapid adaptation to other biomarkers through receptor reprogramming, as evidenced by its parallel success in detecting gut inflammation markers like thiosulfate in IBD patients (Sicard et al., 2014). By overcoming traditional limitations of centralized laboratory diagnostics, this work exemplifies how synthetic biology can bridge precision medicine with global health accessibility, though challenges persist in ensuring sensor stability across diverse microbiota environments.

Shi et al. (2024) developed a bacterial biosensor leveraging the skin commensal bacterium *Staphylococcus epidermidis* to diagnose and treat inflammatory diseases such as psoriasis (Cau et al., 2021; Severn and Horswill, 2022). The biosensor integrates *S. epidermidis* within a dual-network hydrogel matrix composed of gelatin and tapioca starch, mimicking natural biofilm structures to sustain bacterial viability for over 4 days (Xu et al., 2021). Bioinspired double network hydrogels: from covalent double network hydrogels via hybrid double network hydrogels to physical double network hydrogels. This living hydrogel not only adheres conformally to skin but also modulates the immune microenvironment by downregulating pro-inflammatory cytokines (e.g., IL-17, TNF-α) and reducing T-cell infiltration, addressing the root cause of psoriasis. The bacteria’s innate ability to regulate skin homeostasis was enhanced through electrostatic interactions with the conductive polymer, which lowered charge transfer resistance, optimizing electron transfer for real-time monitoring of skin impedance, temperature, and humidity (Zhang P. et al., 2023). A wireless bioelectronic interface enabled on-demand electrical stimulation to control bacterial activity, ensuring biosafety by disinfecting pathogens (e.g., *S. aureus*) via reactive oxygen species generation. The biosensor demonstrated therapeutic efficacy in a psoriasis mouse model, reducing epidermal hyperplasia by 60% and restoring skin microbiota diversity without genetic modification. By synergizing bacterial immunomodulation with bioelectronic sensing, this platform exemplifies the potential of living bacterial systems in precision medicine, offering a drug-free approach to inflammation management while minimizing biohazard risks (Shi et al., 2024). Wang et al. (2022) developed a biosensor based on the electroactive bacterium *Shewanella oneidensis MR-1*, which demonstrates significant potential in medical diagnostics. This system utilizes the bacterium’s intrinsic lactate oxidation capability, where electrons generated from metabolic activity are transferred via outer-membrane cytochrome complexes (e.g., MtrCAB) and riboflavin-mediated extracellular electron transport (EET), enabling label-free lactate detection without genetic engineering (Gurdeep et al., 2021). Electrostatic integration of the conductive polymer poly PMNT enhanced biofilm formation and reduced charge transfer resistance from 226 to 12 Ω, achieving a lactate detection limit of 78 μM in physiological fluids (sweat, urine, plasma). By exploiting the Warburg effect—a hallmark of cancer metabolism characterized by excessive lactate secretion—the biosensor indirectly quantified HeLa, MCF-7, and A549 cancer cells with a sensitivity of 2.9 × 10^4^ cells and an error rate < 10% (Wang et al., 2022). Integration with a flexible wearable platform and wireless signal transmission highlights its clinical applicability for real-time monitoring. Future engineering of *S. oneidensis* to recognize diverse biomarkers (e.g., inflammatory cytokines or pathogens) could expand the utility of living bacterial sensors in precision medicine (Li et al., 2021).

## 5 Challenges and future prospects

Bacterial biosensors exhibit distinct performance advantages over conventional diagnostic technologies, enabled by synthetic biology-driven detection of ultralow-concentration biomarkers in complex biological matrices (Hicks et al., 2020). These systems achieve real-time/near-real-time monitoring of dynamic analyte fluctuations and support multiplexed detection through engineered genetic circuits (Hicks et al., 2020; Table 2). Notably, advanced prototypes integrate diagnostic and therapeutic capabilities, forming closed-loop theranostic systems with feedback-controlled intervention (Jin et al., 2025). However, their medical application faces three primary challenges: functional stability, biosafety, and clinical translation (Riglar and Silver, 2018). Engineered bacteria withstand extreme host environments that involve immune attacks, resource competition with commensal microbiota, and fluctuating physiological conditions, all of which collectively destabilize sensor functionality (Dana et al., 2012). While synthetic genetic circuits provide novel sensing capabilities, they disrupt native metabolic balance, triggering compensatory mutations such as fluorescence reporter inactivation in engineered *E. coli* after serial passages and increasing susceptibility to endogenous signal interference (Sleight and Sauro, 2013; Sleight et al., 2010). Biosafety concerns remain unresolved as conventional suicide switches show limited effectiveness in complex human microenvironments, particularly in intestinal hypoxia zones where occasional bacterial escape may occur (Piraner et al., 2017). Physical encapsulation strategies, though effective in restricting microbial spread, reduce detection sensitivity due to molecular permeability barriers caused by suboptimal material interfaces (Hirota et al., 2017). Clinical translation is further challenged by the lack of standardized validation protocols, rigorous requirements for classifying these systems as “live medical devices” necessitating extensive virulence testing, and difficulties in assessing unpredictable long-term ecological risks. Emerging nanotechnology integration addresses existing limitations through bacterial-nanomaterial co-immobilization strategies. Nanostructure fixation enhances sensitivity by extending cellular electron transfer distances while biomimetic nanocapsules with dynamically tunable pores enable immune evasion and selective molecular permeation (Liu et al., 2024). Concurrently, artificial intelligence-driven platforms revolutionize biosensor development as deep learning models deciphering gene sequence-function correlations enable precise prediction of genetic editing outcomes (Angenent-Mari et al., 2020; Kim et al., 2020). These advancements are combined with microfluidic high-throughput screening to establish Design-Build-Test-Learn (DBTL) closed-loop optimization systems (Berlanda et al., 2021; Orsi et al., 2021). Dynamic evolution systems that simulate *in vivo* pressures accelerate directed bacterial adaptation in microfluidic chips, significantly reducing development timelines. Enhanced biosafety protocols incorporate dual-lock containment mechanisms to reduce escape probability alongside chassis genome minimization strategies for metabolic stability. Regulatory frameworks are evolving through multicenter standardized testing platforms and refined classifications of “live medical device” based on colonization capacity. Researchers are integrating bacterial biosensors with mobile health platforms like smartphones and cloud systems, enabling continuous physiological tracking and remote care delivery via real-time wireless networks (Chang et al., 2017; Raut et al., 2012). While most systems remain experimental, retrospective studies confirm their diagnostic potential in chronic disease management and cancer biomarker detection. Transitioning to clinical practice requires multicenter trials validating sensor stability and specificity in diverse patient populations. Successful validation could translate prototypes into standardized diagnostic modules for healthcare integration. This technological shift promises to enhance detection of complex biomarkers while supporting adaptive treatment protocols through persistent health data streams, ultimately advancing intelligent closed-loop diagnostic-therapeutic systems.

**TABLE 2:** Performance comparison between bacterial biosensors and conventional diagnostic technologies.

Comparison criteria	Bacterial biosensors	Conventional diagnostic technologies (ELISA/PCR/ imaging)
Detection dynamics	Real-time, continuous monitoring	Static detection (relies on single sampling or periodic retesting)
Multi-target capability	Simultaneous multi-target detection via genetic engineering	Primarily single-target detection
Therapeutic integration	Closed-loop detection-treatment systems	Detection function only
Sensitivity	Ultra-low detection limits	Limited sensitivity
Response speed	Rapid response	Time-consuming (e.g., culture-based methods require >24 h; imaging needs complex workflows)
Cost	High R&D cost but low long-term operational costs	High equipment/reagent expenses
Key risks	Biosafety concerns	Reagent stability issues and cross-reactivity
Application scenarios	On-site monitoring, point-of-care diagnostics, personalized medicine	Laboratory-based analysis, standardized protocols
